# The FANTOM web resource: from mammalian transcriptional landscape to its dynamic regulation

**DOI:** 10.1186/gb-2009-10-4-r40

**Published:** 2009-04-19

**Authors:** Hideya Kawaji, Jessica Severin, Marina Lizio, Andrew Waterhouse, Shintaro Katayama, Katharine M Irvine, David A Hume, Alistair RR Forrest, Harukazu Suzuki, Piero Carninci, Yoshihide Hayashizaki, Carsten O Daub

**Affiliations:** 1 RIKEN Omics Science Center, RIKEN Yokohama Institute, 1-7-22 Suehiro-cho Tsurumi-ku Yokohama, Kanagawa, 230-0045 Japan; 2 Institute for Molecular Bioscience, University of Queensland, Brisbane, St Lucia QLD 4072, Australia; 3 The Roslin Institute and Royal (Dick) School of Veterinary Studies, The University of Edinburgh, Roslin, EH259PS, UK; 4 The Eskitis Institute for Cell and Molecular Therapies, Griffith University, QLD 4111, Australia

## Abstract

In FANTOM4, an international collaborative research project, we collected a wide range of genome-scale data, including 24 million mRNA 5'-reads (CAGE tags) and microarray expression profiles along a differentiation time course of the human THP-1 cell line and under 52 systematic siRNA perturbations. In addition, data regarding chromatin status derived from ChIP-chip to elucidate the transcriptional regulatory interactions are included. Here we present these data to the research community as an integrated web resource.

## Abstract 2

The genome-scale data collected by the FANTOM4 collaborative research project are presented as an integrated web resource.

## Background

The Functional Annotation of the Mammalian Genome (FANTOM) consortium is an international collaborative research project initiated and organized by the RIKEN Omics Science Center (OSC; previously Genomics Science Center (GSC)) [1-5] focusing on the characterization and analysis of the transcriptional landscape in mammalian genomes. FANTOM has provided the largest collection of full-length cDNA sequences for several species [1,2,5] and also introduced the cap analysis of gene expression (CAGE) technology to profile transcription initiation [3,5] by 5'-end short tags of mRNAs (CAGE tags). The CAGE tag sequences indicate the location of transcriptional starting sites (TSSs) [6]. Our efforts uncovered that a substantial part of the mammalian genome is transcribed and that the number of TSSs is substantially larger than previously expected. Additionally, a large amount of messenger-like RNAs that do not encode proteins was detected [3-5].

The fourth round, FANTOM4, aimed at the elucidation of the transcriptional regulatory network controlling the differentiation of a human macrophage-like cancer cell line [7]. As our knowledge about transcriptional regulatory interactions is still far from complete, in-depth delineation of transcriptional regulatory input and output in different conditions of a biological system is required, in particular, transcription factor binding to the genome and chromatin modification as regulatory inputs, and transcriptional initiation activity and mRNA abundance as output. Comprehensive characterization of these aspects in a mammalian system has not previously been attempted. For FANTOM4, we focused on the differentiation of THP-1 monoblastic leukemia cells when stimulated with phorbol myristate acetate (PMA). Upon PMA treatment, THP-1 cells switch from a proliferating monoblast-like state (round cells growing in suspension) into a differentiated monocytic-like state (adherent cells with a flattened morphology that cease to proliferate).

To understand the transcriptional changes that facilitate this state transition, we measured mRNA expression changes using microarrays and used next-generation deepCAGE tag sequencing to monitor the dynamics of transcriptional initiation at the promoter level. We then used bioinformatic approaches to predict the transcription factors that regulate these promoters. To test these predictions, we carried out small interfering RNA (siRNA) knockdown of 52 transcription factors expressed in THP-1 cells and monitored their effect on the predicted targets and all other genes using microarrays. The majority of these knockdowns focus on transcription factors that are themselves transcriptionally repressed in response to PMA. Finally, we complemented these datasets with chromatin immunoprecipitation with microarray (ChIP-chip) for several key factors and markers of active transcription (the macrophage-specific factor PU.1, general transcription factor SP1, histone H3 lysine 9 (H3K9) acetylation and RNA polymerase II).

The data obtained will serve as an essential resource for further analyses for the scientific community. Thus, we accumulate all of these data into a single web resource and make it publicly accessible. An easy-to-use graphical interface facilitates the integrated visual inspection for the scientific community and a collection of all underlying data files enables further bioinformatics analyses. We complemented the resource by incorporating CAGE data previously obtained from a wide-range of cell types. This public resource offers one of the most extensive perspectives of promoter activities to date. Here, we describe the web resource with the experimental details.

## Results

### High-resolution and genome-wide profile of transcriptional regulatory input and output in the differentiation time course

**Figure 1:**
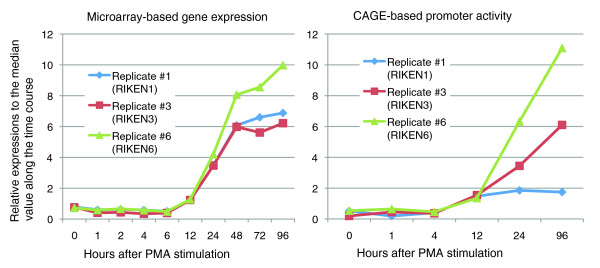
*CD11b *activity. Microarray-based expression and CAGE-based promoter activities of *CD11b*, a monocytic marker gene.

We performed a wide range of genome-wide experiments for the THP-1 cells (Table 1). To ensure the consistency and comparability of the data taken, all experiments were based on cells that were cultured and cultivated from one initial cell batch (Additional data file 1). The differentiation time course was analyzed in three independent experiments (biological replicates). The expression of all genes was monitored over the time course with microarrays to ensure that the known THP-1 specific marker genes responded correctly. For example, a monocytic marker gene, *CD11b *(*ITGAM*), is up regulated in all three biological replicates (Figure 1).

**Table 1:** Data content of the FANTOM web resource

Cell conditions and biological replicates	Type of experiments	Track names in the genome browser
THP-1 differentiation time course × 3	CAGE	TSS positions
		Promoters
		Promoter regions
THP-1 differentiation time course × 3	Illumina microarray	Illumina biological replicates
THP-1 systematic perturbation (52 transcription factors knocked down with siRNA) × 3	Illumina microarray	Illumina knock-down siRNA series
THP-1 before and after differentiation × 2	H3K9Ac ChIP-chip with whole genome tiling array	H3K9 by whole tiling array
THP-1 before and after differentiation × 1	RNA polymerase II ChIP-chip with whole genome tiling array	RNA polymerase II H3K9 by whole tiling array
THP-1 before and after differentiation × 2	PU.1 ChIP-chip with promoter tiling array	PU.1 promoter array
THP-1 before and after differentiation × 2	SP1 ChIP-chip with promoter tiling array	SP1 promoter array
THP-1 differentiation time course × 1	Small RNA sequencing	ShortRNA cluster, shortRNA sequence
THP-1 differentiation time course × 2	qRT-PCR for transcription factors	Transcription factor qRT-PCR
Human 127 RNA samples (including the differentiation time courses)	CAGE	Tag cluster
Mouse 206 RNA samples (including the differentiation time courses)	CAGE	Tag cluster

We also profiled TSSs by sequencing of 5'-end tags of mRNAs, CAGE tags, employing the 454 next-generation sequencer [8]. For six time points after PMA stimulation of THP-1 cells, we profiled all three biological replicates independently, resulting in 24 million CAGE tags. We defined 1.9 million individual TSSs (level 1 promoters) giving single base pair resolution of transcriptional initiation; 30,000 promoters (level 2 promoters) contained neighboring TSSs with similar expression profiles over the time course (TSSs were merged to form level 2 promoters); 15,000 promoter regions (level 3 promoters) contained continuous promoters separated by no more than 400 bp distance on the genome (level 3 promoters). We assigned expression profiles according to all of the three levels of transcription initiation based on CAGE data so that promoter activities between independent promoters as well as between the biological replicates can be compared [7]. In general, there was a strong correlation between data from microarray analysis and CAGE profiling. For example, the profiled promoter activity of the marker gene *CD11b *shows up-regulation in all three biological replicates, which is consistent with the observed microarray-based gene expression (Figure 1). However, in some genes, such as PU.1, there is divergent regulation of independent promoters [7].

For two time points, in the beginning and at the end of the time course, whole genome ChIP-chip experiments for the epigenetic mark of H3K9 acetylation and for the interaction of RNA polymerase II with DNA were performed. Both show substantial (approximately 80%) overlap with the promoter sets defined by deepCAGE [7], while there is no complete overlap as shown in a previous study [9]. Reliable expression over the biological replicates as well as the reasonable coincidence of TSSs and the chromatin status demonstrate the reproducibility and reliability of the experimental data. Additional ChIP-chip experiments for two transcription factors known to be important in macrophage differentiation, SP1 and PU.1, were performed using promoter tiling arrays for two biological replicates. Both factors bind to the promoter region of *CD11b*, which is consistent with previous studies [10,11].

### Transcriptional regulatory network analysis and 52 systematic siRNA perturbation experiments

We predicted transcriptional regulatory interactions employing a hybrid approach of: transcription factor binding site (TFBS) predictions within evolutionarily conserved regions that are proximal to the CAGE defined promoter regions (-300 bp to +100 bp) using TFBS matrices; and microarray derived gene expression. To validate the predicted interactions, we performed large-scale siRNA perturbation knockdown experiments for 52 transcription factors that are expressed in the undifferentiated state, including the two transcription factors used in the ChIP-chip experiments, SP1 and PU.1. We monitored the effects on gene expression with microarrays at 48 h after transfection. All transfection and subsequent microarray experiments were conducted in biological triplicates. We observed down-regulation of the *CD11b *gene upon knockdown of SP1 and PU.1, which is consistent with their binding to the *CD11b *promoter region as well as with previous studies [10,11].

Interestingly, our large-scale perturbation study revealed that knocking down of Myb induced the expression of *CD11b *and many other genes that are up-regulated during THP-1 differentiation, indicating that Myb can work directly or indirectly as a transcriptional repressor [7]. The expression of Myb is itself rapidly repressed in response to PMA.

### Quantitative RT-PCR for transcription factors, small RNA sequencing, and update of CAGE data

Additional data, in-depth sequencing of small RNA and large-scale quantitative RT-PCR (qRT-PCR) of 2,000 transcription factors were performed subsequently to complement the analysis. The deep sequencing of small RNAs, ranging from 10 to 82 nucleotides, uncovered a novel class of transcription initiation-associated short RNAs with a length around 18 nucleotides [12]. Moreover, four novel microRNAs were found together with general aspects of microRNA expression during THP-1 differentiation (manuscript under review). The viewer also incorporates large-scale qRT-PCR expression profiling of 2,000 transcription factors along the time course for two biological replicates and gives detailed expression levels for these transcription factors, which are generally difficult to capture with conventional microarrays [13]. Additional ongoing experiments, including data obtained from primary monocytes and monocyte-derived macrophages, will be continuously incorporated into this web resource as soon as they are published.

In addition to the above data focusing on THP-1, we updated the genome mapping of the 5 million FANTOM3 CAGE tags obtained from 41 human samples [3]. We incorporated other published human RIKEN CAGE tags as well, resulting in a total of 29 million mapped tags from 127 human RNA samples. Genomic mapping of previously published RIKEN mouse CAGE tags were updated to the latest genome assembly, mm9 (NCBI build 37), resulting in 11 million mapped tags from 206 mouse RNA samples. All of these CAGE data were processed in a uniform way in terms of alignment to the genome and aggregation into promoters as 'tag clusters' consistent with previous work [3,14].

### The database and its interface

**Figure 2:**
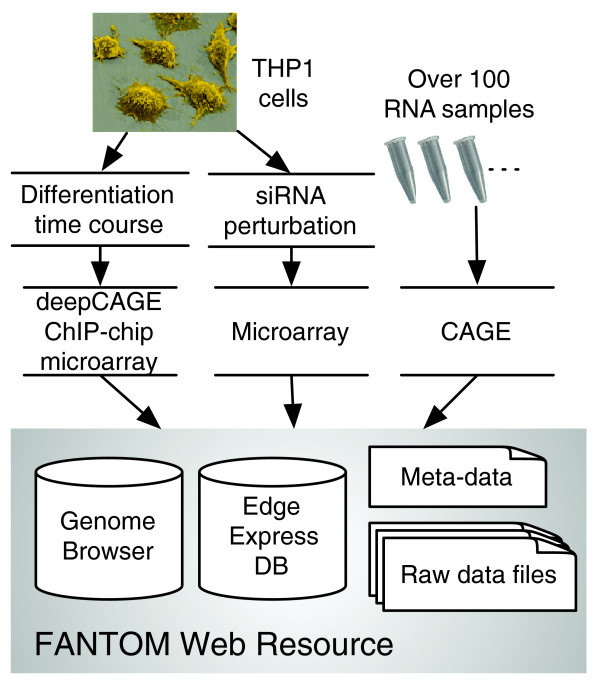
Overview of the FANTOM web resource. The FANTOM4 data and the other CAGE tags are collected in the FANTOM web resource, which consists of the genome browser interface and data files.

**Figure 3:**
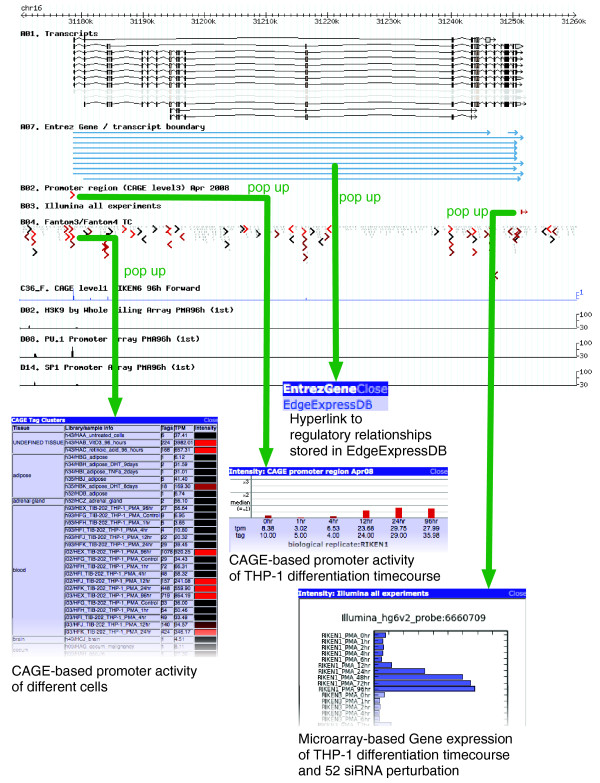
Genome browser interface. Details about the items displayed in the genome browser can be inspected with several parallel pop-up windows. Microarray-based gene expression is displayed in a horizontal bar plot. Promoter activities along with THP-1 time course and different tissues are displayed as a vertical bar plot and a heatmap table, respectively.

All data are accumulated into a single web resource consisting of two databases, the Genome Browser and the EdgeExpressDB [15], as well as a repository for data downloads (Figure 2). The genomic data derived from our experiments are displayed via a genome browser interface (generic genome browser, GBrowse) [16] enabling the inspection of any locus of interest. The browser was configured to show multiple experimental results and multiple conditions at the same time in small 'pop-up' windows within one single browser window (Figure 3), which is implemented with the coolTip library [17]. For example, a mouse click on '*CD11b *promoter' produces a small window presenting quantitative promoter activity, in particular, up-regulation of this promoter in THP-1 cells in response to PMA. A mouse click on the microarray probe corresponding to this gene produces a small window depicting its expression, which also shows increased abundance of the mRNA of CD11b. The parallel display of these two windows enables the examination of the relationship between promoter activities identified by CAGE and gene expressions measured by the microarray (Figure 3). Tissue-wide promoter activities derived from CAGE tags sequenced before the FANTOM4 project are available in the same interface (Figure 3), making it possible to examine tissue- or cell-specific expression.

The FANTOM4 EdgeExpressDB [15] facilitates the inspection of regulatory interactions and expression profiles in the same context, which is impossible using the genome browser only. It stores predicted and validated regulatory relationships as well as all of the corresponding expression profiles. The EdgeExpressDB and the genome browser are cross-referenced with hyperlinks (Figure 3). The two interfaces visualize complementary aspects of the same complex data.

In addition to the graphical user interface, we have prepared an application interface (API) for direct access using the DAS protocol [18], which is widely used to exchange genome annotation information. The available tracks and their entry points are described in Table 1 and Additional data file 2.

### Standardized meta-data describing large-scale multi-dimensional experiments

A wide-range of large-scale experiments with several replicates was performed for the THP-1 differentiation in response to PMA stimulation. Analyses and findings are being reported in a series of separate publications, each of them employing parts of the complex data. Understanding interconnections between experiments and the data as a whole is challenging. To describe the whole set of experiments in a consistent way, we employed the MAGE/ISA-tab file format [19,20], a standard format to describe experimental details (available in the 'Download' section of the FANTOM web resource). The file connects all experiments on the level of cell cultivation and biological replicates even if they were used in different analyses. The experimental steps described in the file are visualized with SDRF2GRAPH [21] to support intuitive understanding of the complex experimental steps (Additional data file 1). These meta-data files help to document the data structure of the FANTOM4 project and support its use and biological interpretation. While the FANTOM3 data were centered around the CAGE technology [14], we now explicitly describe the relationships between all types of the experiments in a comprehensive way based mainly upon genomic location.

## Discussion

We accumulated a wide-range of experimental data focusing on one particular biological system, THP-1, into a unique resource to promote the understanding of transcriptional regulation on a genome-scale. The number of CAGE tags derived from the THP-1 cell line is approximately fivefold higher than the number of all human CAGE tags derived from 41 RNA samples taken in FANTOM3 [3]. In contrast to the relatively shallow sequencing of the FANTOM3 CAGE data, the FANTOM4 deepCAGE data sample sufficient tags from any one library to give a quantitative picture of expression of transcripts initiated from individual TSSs and promoters. The correlation with mRNA levels measured by other methods is imperfect mainly because many genes have more than one promoter [5].

An independent collection of 5'-end reads of human transcripts, DBTSS [22], comprises 18 million reads. With its latest update, 100 million reads under RNA interference experiments have been added [23]. The FANTOM4 data consist of a similar order of 5'-end reads of mammalian transcripts (40 million reads in total; 29 million from human and 11 million from mouse), including sampling along the differentiation time course enabling monitoring of the important perspective of transcriptional initiation activities and their transitions. Furthermore, our data integrate a wide range of experimental data targeting complementary aspects of the very same biological system, such as TSS identification in a quantitative way, expression profiling by microarrays and large-scale qRT-PCR for virtually all transcription factors, epigenetic modifications of chromatin, large-scale siRNA perturbation experiments, and short RNA sequencing.

The ENCODE database [24] collected a wide-range of experiments, where the analyses were mainly limited to just 1% of the genome. While genome-wide experiments have been included increasingly [25], the FANTOM web resource provides large-scale siRNA perturbation experiments addressing transcriptional regulatory interactions, combined with a collection of truly genome-wide experiments. Our resource offers a unique basis to investigate the transcriptional machinery by providing the genome-wide high-resolution data to address questions about transcriptional regulatory interactions.

## Conclusions

The presented FANTOM web resource updates and integrates data and analysis results of previous FANTOM activities as well as of the ongoing FANTOM4 activity. Containing 40 million CAGE tags (29 million from 127 human RNA samples and 11 million tags from 206 mouse RNA samples), the FANTOM web resource is one of the most complete resources of TSSs available. The focus of the FANTOM4 project on the comprehensive and detailed characterization of the differentiation of THP-1 cells makes the FANTOM web resource the largest experimental data repository for the very well studied THP-1 cell line model system.

We are continuously producing and collecting CAGE data for various organisms and experimental conditions and successively integrate it into the FANTOM web resource. Our explicit goal is to making this web resource the central repository for CAGE data in the world.

## Materials and methods

### Cell culture and RNA extraction

The THP-1 cell line was subcloned by limit dilution and one clone (clone 5) was selected for its ability to differentiate relatively homogeneously in response to PMA. THP-1 cells were used for all subsequent experiments. THP-1 cells were cultured in RPMI, 10% fetal bovine serum, penicillin/streptomycin, 10 mM HEPES, 1 mM sodium pyruvate and 50 μM 2-mercaptoethanol. THP-1 was treated with 30 ng/ml PMA (Sigma, St Louis, MO, USA) over a time course of 96 h. Total cell lysates were harvested in TRIzol reagent (Invitrogen, Carlsbad, CA, USA) at each time point. Undifferentiated cells were harvested in TRIzol reagent at the beginning of the PMA time course. Total RNA was purified from TRIzol lysates according to the manufacturer's instructions.

### DeepCAGE

The preparation of the CAGE library from total RNA was a modification of methods described by Shiraki *et al. *[26] and Kodzius *et al. *[6], adapted to work with the 454 Life Sciences sequencer. The sequenced CAGE tags were firstly mapped onto the latest genome assemblies, hg18 (NCBI build 36.1), using an in-house developed program, nexAlign, and post-processed to consider CAGE tags mapped to multiple loci [27]. The mapped tags were then clustered into three different levels of detail: on individual TSSs giving single base pair resolution for each CAGE tag; on the level of promoters joining neighboring TSSs with similar expression profiles over the time course; and on the level of promoter regions containing continuous promoters separated by a distance of 400 bp on the genome. This computation was performed subsequent to expression normalization to enable comparisons between the biological replicates, resulting in a set of TSS positions, promoters and promoter regions with genomic locations and normalized expression for each time point in the differentiation time course.

### Illumina microarray analysis

THP-1 samples were identical to those used for deepCAGE libraries, and RNA was purified for expression analysis by RNeasy columns (Qiagen, West Sussex, UK), FastPure RNA Kit or TRIzol (TAKARA BIO, Otsu, Shiga Japan). RNA quality was checked using the Nanodrop (Nanodrop, Wilmington, DE, USA) and Bioanalyser (Agilent, Santa Clara, CA, USA). RNA (500 ng) was amplified using the Illumina TotalPrep RNA Amplification Kit, according to the manufacturer's instructions. cRNA was hybridized to Illumina Human Sentrix-6 bead chips Ver.2, according to standard Illumina protocols. Chip scans were processed using Illumina BeadScan and BeadStudio software packages and summarized data were generated in BeadStudio (version 3.1).

### ChIP on chip analysis

THP-1 cells were cross-linked with 1% formaldehyde for 10 minutes and cells were collected by centrifugation and washed twice in cold 1× phosphate-buffered saline. The cells were sonicated for 5-7 minutes with a Branson 450 Sonicator to shear the chromatin (Branson Ultrasonic, Danbury, CT, USA). Complexes containing DNA were immunoprecipitated with antibodies against H3K9Ac (07-352; Upstate Biotechnology, Lake Placid, NY, USA), PU.1 (T-21; Santa Cruz Biotechnology, Santa Cruz, CA), SP1 (07-645; Upstate), and RNA Polymerase II (8WG16; Abcam, Cambridge, MA, USA). The immunoprecipitated sample was incubated with magnetic beads/Protein G (Dynal, Oslo, Norway) for 1 h at 4°C followed by washing. The complexes were eluted from the magnetic beads by addition of 1% SDS and 100 mM NaHCO_3_. Beads were vortexed for 60 minutes at room temperature. The supernatants were incubated for 3.5 h at 65°C to reverse the cross-links, and incubated with RNaseA, and then proteinase K, followed by a phenol:chloroform:isoamyl alcohol extraction and ethanol precipitation to recover the DNA. Immunoprecipitated DNA was amplified by either linker-mediated PCR or *in vitro *transcription followed by synthesis of double-strand cDNA. Amplified DNA was end-labeled with biotin-ddATP and was hybridized to Affymetrix whole genome tiling for Polymerase II (GeneChip Human Tiling 1.0R Array) and H3K9Ac (GeneChip Human Tiling 1.0R Array), and promoter arrays (GeneChip Human Promoter 1.0R Array) for PU.1 and SP1.

### siRNA perturbation experiments

THP-1 cells were seeded in 6 cm dishes at a density of 1 × 10^6 ^cells/dish for transfection. Transfection was performed with 1.6 μg/ml (final concentration) of Lipofectamine 2000 (Invitrogen) and 20 μM (final concentration) of stealth siRNA (Invitrogen) or 20 μM (final concentration) of pre-microRNA (Ambion, Austin, Tx, USA, or Nihon-shinyaku, Kyoto, Japan) by reverse transfection protocol in accordance with the manufacturers' instructions. Total RNA for Illumina microarray analysis was extracted 48 h after transfection, using the FastPure RNA kit (TAKARA BIO, Ohtsu, Shiga, Japan) in accordance with the manufacturer's instructions. All microarray experiments were conducted in biological triplicate.

### Resource availability

The graphical user interface, DAS API, the original GFF files of the genome browser, and all the additional data files are publicly available at [28].

## Abbreviations

API: application interface; CAGE: cap analysis of gene expression; ChIP-chip: chromatin immunoprecipitation with microarray; FANTOM: Functional Annotation of the Mammalian Genome; H3K9: histone H3 lysine 9; PMA: phorbol myristate acetate; qRT-PCR: quantitative RT-PCR; siRNA: small interfering RNA; TFBS: transcription factor binding site; TSS: transcriptional starting site.

## Authors' contributions

HK, JS, and ARRF organized the data stored in the database. HK and ML configured and set up the genome browser database. JS, ARRF, and AW designed, implemented and set up the FANTOM4 EdgeExpressDB instance. SK integrated all the available CAGE tags as tag clusters. HS, YH, PC, KI and DH constructed the original datasets. HS, PC, YH, and CD were involved in conceptualization and supervision. HK wrote the manuscript, and ARRF, PC, and CD improved the manuscript.

## Additional data files

The following additional data are available with the online version of this paper: a figure showing the experimental steps of cell and RNA preparations (Additional data file 1); an extended version of Table 1, which includes DAS sources and GFF files for the data shown in the genome browser (Additional data file 2).

## Supplementary Material

Additional data file 1 An investigation design graph of RNA preparation showing the cell batches are prepared from the same original cells, treated with several conditions, and RNA is extracted. These steps are described in the MAGE/ISA-tab file as meta-data, which include all of our experimental details, and the content is visualized as an investigation design graph by SDRF2GRAPH [20]. Click here for file

Additional data file 2 An extended version of Table 1, which includes DAS sources and GFF files for the data shown in the genome browser. Click here for file
